# *Hoxa9*/*meis1*-transgenic zebrafish develops acute myeloid leukaemia-like disease with rapid onset and high penetrance

**DOI:** 10.1098/rsob.220172

**Published:** 2022-10-26

**Authors:** Wei Wang, Hongji Li, Mengling Huang, Xue Wang, Wei Li, Xiaoqing Qian, Lili Jing

**Affiliations:** ^1^ Engineering Research Center of Cell and Therapeutic Antibody, Ministry of Education, Pharm-X Center, School of Pharmacy, Shanghai Jiao Tong University, Shanghai 200240, People's Republic of China; ^2^ Core facility and technical service center, School of Pharmacy, Shanghai Jiao Tong University, Shanghai 200240, People's Republic of China; ^3^ School of Biomedical Engineering, Shanghai Jiao Tong University, Shanghai 200240, People's Republic of China

**Keywords:** *hoxa9*, *meis1*, acute myeloid leukaemia, transgenic zebrafish model, myeloid malignancy

## Abstract

*HOXA9* and *MEIS1* are co-expressed in over 50% of acute myeloid leukaemia (AML) and play essential roles in leukaemogenesis, but the mechanisms involved are poorly understood. Diverse animal models offer valuable tools to recapitulate different aspects of AML and link *in vitro* studies to clinical trials. We generated a double transgenic zebrafish that enables *hoxa9* overexpression in blood cells under the draculin (*drl*) regulatory element and an inducible expression of *meis1* through a heat shock promoter. After induction, Tg(*drl*:*hoxa9*;*hsp70*:*meis1*) embryos developed a preleukaemic state with reduced myeloid and erythroid differentiation coupled with the poor production of haematopoietic stem cells and myeloid progenitors. Importantly, most adult Tg(*drl*:*hoxa9*;*hsp70*:*meis1*) fish at 3 months old showed abundant accumulations of immature myeloid precursors, interrupted differentiation and anaemia in the kidney marrow, and infiltration of myeloid precursors in peripheral blood, resembling human AML. Genome-wide transcriptional analysis also confirmed AML transformation by the transgene. Moreover, the dihydroorotate dehydrogenase (DHODH) inhibitor that reduces leukaemogenesis in mammals effectively restored haematopoiesis in Tg(*drl*:*hoxa9*;*hsp70*:*meis1*) embryos and improved their late survival. Thus, Tg(*drl*:*hoxa9*;*hsp70*:*meis1*) zebrafish is a rapid-onset high-penetrance AML-like disease model, which provides a novel tool to harness the unique advantages of zebrafish for mechanistic studies and drug screening against *HOXA9*/*MEIS1* overexpressed high-risk AML.

## Graphical abstract

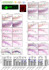

## 1. Introduction

Acute myeloid leukaemia (AML) is an aggressive neoplastic disease characterized by differentiation arrest and uncontrolled proliferation of immature cells of the myeloid lineage [1]. It is a highly heterogeneous and complex disease. Traditional chemotherapy using cytotoxic agents has been the mainstay of AML treatment for decades, with a 5-year survival rate of 30–35% [2]. Recent studies have identified important genetic alterations in AML and have led to development of new drugs targeting those specific mutations [3,4]. Since 2017, several targeted therapies have been approved for AML treatment, such as FMS-like tyrosine kinase 3 (FLT3) inhibitor for AML patients with FLT3 mutations and isocitrate dehydrogenase (IDH) 2 inhibitor for AML with IDH2 mutations [5,6]. However, for AML of unknown aetiology, there is still an urgent need to develop novel treatments.

AML is an intricate disease, and cultures of single-cell line systems can never recapitulate the complexity of the disease [7]. Diverse animal models have been used to study the mechanisms responsible for the cellular and molecular pathologies of AML and for drug screening. Among these models, zebrafish (*Danio rerio*) has proven to be a useful model for studying haematopoietic regulation and blood disorders [8]. The zebrafish model affords many advantages, such as high fecundity, rapid external embryo development, numerous fluorescent reporter lines and genetic tractability [9]. In addition, the cellular processes and genetic programmes of haematopoiesis are highly conserved between zebrafish and mammals.

A series of myeloid leukaemia models have been established using zebrafish [10–12]. Most of these models are based on exogenous expression of human AML fusion oncogenes or genetic mutations. These mutations in zebrafish often disrupt embryonic haematopoiesis, resulting in distinct phenotypes such as expansion of the myeloid compartment and anaemia. These embryonic models partially recapitulate the symptoms observed in mice and humans, which offer a fast tool useful for mechanistic studies and drug discovery. However, the embryonic models do not represent a full adult-arising leukaemia. Unfortunately, the haematological abnormalities that present early in these models are often embryonic lethal, which precludes studies of adult leukaemia [9]. Alternatively, the transgene may survive into adulthood, but usually develops into leukaemia with long latency and low penetrance, making further study inconvenient. For example, Tg(*NUP98*-*HOXA9*) zebrafish used a Cre-LoxP system to drive inducible expression of human *NUP98*-*HOXA9* in blood cells, which circumvented early embryonic death. About 23% of the transgenic fish developed preleukaemia myeloproliferative neoplasms (MPNs) by 2 years after a long latency period [13]. This has become a limitation of the zebrafish system for myeloid disease research.

In nearly 80% of AML cases, HOXA9 is upregulated, and it is one of the most important predictors of treatment failure by traditional chemotherapy and radiation therapy [12,14,15]. A variety of upstream genetic alterations can lead to deregulation of HOXA9 [16]. HOXA9 is also directly involved in recurrent translocations, resulting in fusion transcripts, such as NUP98-HOXA9 oncogene [17]. Hoxa9 overexpression alone in mouse bone marrow cells leads to a myeloproliferative disorder that progresses to AML around 7 months, suggesting the requirement for additional genetic events prior to AML [17]. HOXA9 is also often co-overexpressed with other cofactors. One of the cofactors, myeloid ecotropic viral integration site 1 (MEIS1) is a three-amino acid-loop-extension homeodomain protein [18,19]. The co-overexpression of HOXA9 and MEIS1 is observed in over 50% of human AML [20,21]. Overexpress *Hoxa9*/*Meis1* in murine primary bone marrow cells, when transplanted into syngeneic mice, induced AML rapidly in 49–75 days [22]. In fact, *Hoxa9*/*Meis1* combination is one of the most potent AML-inducing oncogenes that induce the fastest occurrence of leukaemia [23,24]. The mouse *Hoxa9*/*Meis1* AML model is now widely used to study the pathogenesis of AML and to test anti-leukaemic therapies. However, there is currently no zebrafish *hoxa9*/*meis1* model. The construction of a zebrafish *hoxa9*/*meis1* model would expand our ability to study AML and help to develop new drugs.

In this study, we generated a double transgenic zebrafish harbouring *hoxa9*/*meis1* under the control of zebrafish *draculin* (*drl*) haematopoietic cell regulatory element [25] and heat shock-inducible *hsp70* promoter [26]. In embryos, enforced *hoxa9*/*meis1* expression led to defective myeloid, erythroid and lymphoid development coupled with poor haematopoietic stem cells (HSCs) output. At the age of 3 months, most Tg(*drl*:*hoxa9*;*hsp70*:*meis1*) showed expansion of immature myeloid precursors and anaemia in the kidney marrow (KM) and significant infiltration of myeloid precursors in the peripheral blood (PB), similar to AML in mammals. We propose that Tg(*drl*:*hoxa9*;*hsp70*:*meis1*) zebrafish is a unique tool to study the related AML pathogenesis and treatment.

## 2. Materials and methods

### 2.1. Zebrafish maintenance and embryos handling

Zebrafish (*Danio rerio*) were raised and maintained in a circulating system (28.5°C, pH 7.3, conductivity of 400 mS cm^−1^ and 14 h light/10 h dark cycle) and fed two times per day. Embryos were maintained in E3 medium (5 mM NaCl, 0.17 mM KCl, 0.33 mM CaCl2 and 0.33 mM MgSO4). Wild-type (WT) zebrafish of the AB strain were used in the study.

### 2.2. Generation of transgenic zebrafish and heat shock treatment

The full-length CDS of *hoxa9* was tagged with 6xmyc and cloned after the *drl* regulatory element in a vector that also contained *∂*-crystallin::Venus (CV) reporter gene. Full-length CDS of *meis1b* (*meis1* hereafter) together with 2A-GFP was cloned downstream of zebrafish *hsp70* promoter in a *pcs2* expression vector [27]. These plasmids were separately injected into WT zebrafish embryos at one-cell stage to generate stable Tg(*drl*:*hoxa9*) or Tg(*hsp70*:*meis1*). Tg(*drl*:*hoxa9*) was crossed with Tg(*hsp70*:*meis1*) to obtain a double transgene fish Tg(*drl*:*hoax9*;*hsp70*:*meis1*). Tg(*drl*:*hoax9*;*hsp70*:*meis1*) was identified by the expression of GFP in the whole embryos after heat shock induction at 1 dpf and the expression of GFP in the eyes at 3 dpf. To induce expression of Tg(*hsp70*:*meis1*), embryos were heat induced at 38.5°C for 1 h. For repeated heat shock inductions, embryos were heat induced at 38.5°C for 1 h every 3 days starting at 24 hpf. These heat shock treatments had little effect on the health of the zebrafish.

### 2.3. Whole-mount *in situ* hybridization and probe synthesis

Whole-mount *in situ* hybridization (WISH) was performed as previously described [28]. The digoxigenin-UTP labelled oligonucleotides were synthesized using an *in vitro* transcription system. *cmyb*, *mpx*, *mfap4*, *gata1*, *rag1* and *hbbe*1 probes were synthesized from linearized plasmids with T7 polymerase. *runx1* probe were synthesized from PCR template with T7 polymerase. The PCR template was amplified from zebrafish cDNA with *runx1* specific primers, which were 5′-CGCCTCCCTCCACCACCCTGA-3′ and 5′-GCGTAATACGACTCACTATAGGGCTGGTTGGCGAACTGCTGTG-3′. At the desired time points, embryos were fixed overnight in 4% paraformaldehyde (PFA) at 4°C, bleached and dehydrated in methanol at −20°C. Further processing of embryos was conducted according to the protocol previously published [24]. The stained embryos were imaged under SZX16 stereomicroscope or BX53 microscope (Olympus, Japan).

### 2.4. Neutral red staining

Embryos were treated with 0.003% N-phenylthiourea PTU (Sigma-Aldrich, St. Louis, MO, USA) between 10 and 24 hpf to prevent pigment formation. Staining of macrophages in live embryos was obtained by incubating embryos in 2.5 µg ml^−1^ neutral red (Sangon Biotech, China) in E3 medium containing PTU at 28.5°C in the dark for 6–8 h according to the procedures in previous studies [29]. The stained embryos were imaged under SZX16 stereomicroscope.

### 2.5. Sudan black staining

Embryos were fixed overnight in 4% PFA at 4°C and washed three times in PBS. Embryos were then stained with Sudan black solution (0.18% stock diluted 1 : 5 in 70% ethanol, 0.1% phenol) for 30 min to 5 h. After neutrophil granules have taken up the stain, embryos were treated with a series of 5 min washes, and a single wash in 70% ethanol followed by a single wash of a 1 : 1 ratio of 70% ethanol and PBS. The embryos are finally washed in PBS. The stained embryos were imaged under SZX16 stereomicroscope.

### 2.6. Benzidine staining

Embryos were treated with 0.003% N-phenylthiourea PTU between 10 and 24 hpf to prevent pigment formation. Embryos were incubated in benzidine staining solution (4 ml 5 mg ml^−1^ benzidine stock prepared in methanol, 4.966 ml ddH_2_O, 33.4 µl 3 M NaOAC and 200 µL 30% H_2_O_2_) for 30 min in the dark. After washed with PBST (PBS containing 1% Tween20), embryos fixed in 4% PFA overnight and imaged under stereomicroscope.

### 2.7. Western blot

Embryos were lysed in RIPA cell lysis buffer (Beyotime, Haimen, China) supplemented with PMSF (1 mM) for 30 min at 4°C with occasional rocking. After centrifugation, the supernatants were harvested and boiled for 10 min. The total protein concentration was determined by the BCA assay kit (Beyotime, Haimen, China). Embryos lysates were loaded onto SDS-PAGE and transferred to PVDF membranes. The antibodies used were as follows: anti-β-actin antibody (1:500, Proteintech, 66009-1, China) and anti-meis1 antibody (1:200, Santa Cruz, sc-101850, USA).

### 2.8. Antibody staining of the embryos

The Tg(*drl*:*hoxa9*) embryos at 28-somite stage were fixed in 4% PFA plus 1% DMSO in 0.1 M phosphate buffer (PB). Embryos were then washed three times using PB and dehydrated in MeOH at −20°C, followed by pre-chilled acetone treatment for 7–20 min at −20°C. Embryos were then washed with incubation buffer (IB, 0.1 M PB buffer containing 0.2% BSA and 0.5% Triton-X) three times. After that, embryos were incubated with anti-myc (Proteintech, 60003-2-1g, China) overnight at 4°C, washed with IB and then incubated with 594-conjugated goat anti-mouse IgG (H + L) secondary antibody (Proteintech, SA00013-3, China). The stained embryos were imaged under a MVX10 fluorescence microscope (Olympus, Japan).

### 2.9. Cytology analysis of whole kidney marrow and peripheral blood of adult fish

The adult fish KM was dissected and placed into FACS buffer (ice cold 0.9X phosphate-buffered saline containing 5% fetal calf serum). The single haematopoietic cells from KM were generated by pipetting and filtration through 40 µm filters. PB was obtained by puncturing the tail using a scalpel and collected using micropipette tips coated with heparin. Blood samples were immediately placed into FACS buffer for further cytological analysis. The whole KM and PB were collected from each fish, and the fish were then euthanized. The single-cell suspensions were cytocentrifuged at 300 rpm for 3 min with Cytospin 4 (Sigma Biomolecules, St Louis, MO, USA). The slides were double stained by May-Grünwald and Giemsa (Sigma-Aldrich, St Louis, MO, USA) for morphological analysis and cell counting according to the manufacturer's protocol.

### 2.10. Tissue sections, histology and immunohistochemistry

Whole adult zebrafish were euthanized with 2 mg ml^−1^ Tricaine (Sigma-Aldrich, St Louis, MO, USA), fixed in 4% PFA and embedded in paraffin, and 5 µm serial sections were obtained and stained with haematoxylin and eosin (H&E) or periodic acid-Schiff (PAS) according to the standard procedures [30].

### 2.11. Real-time qPCR

Total RNA was extracted from embryos using Trizol reagent (Thermo Fisher Scientific, Waltham, MA, USA). The cDNA was reverse transcribed using Hifair II first-strand cDNA synthesis supermix for qPCR (1123ES60, Yeasen Biotechnology, shanghai, China). The qPCR analysis was performed with Hieff qPCR SYBR Green Master Mix (11203ES08, Yeasen Biotechnology, shanghai, China) in a StepOnePlus real-time PCR machine (Applied Biosystems, USA). Each target gene was calculated using the 2^−ΔΔCT^ method. The primers for target genes are listed in electronic supplementary material, table S1.

### 2.12. Analysis of genome-wide transcription changes

To evaluate genome-wide gene expression changes, the KM cells were harvested from adult WT or Tg(*drl*:*hoxa9*;*hsp70*:*meis1*) zebrafish (about 5 months old). The blood cells from three KMs were mixed into one sample and the total RNA was extracted using Trizol. Three biological triplicates were used for each group. The total RNA quantity and purity were analysed by Bioanalyzer 2100 and RNA 6000 Nano LabChip Kit; high-quality RNA samples with RIN number greater than 7.0 were used to construct sequencing library. We performed the 2 × 150 bp paired-end sequencing (PE150) on an Illumina Novaseq 6000 (LC-Bio Technology CO., Hangzhou, China) following the vendor's recommended protocol.

### 2.13. RNA-seq analysis

Reads obtained from the sequencing machines were further filtered by Cutadapt (version:1.9) to get high-quality clean reads. Then, we aligned reads of all samples to the research species reference genome using HISAT2 (version:2.0.4) package, which initially remove a portion of the reads based on quality information accompanying each read and then maps the reads to the reference genome (GRCz11, from the Ensembl genome database project). The mapped reads of each sample were assembled using StringTie (version:1.3.4d) with default parameters. Next, all transcriptomes from all samples were merged to reconstruct a comprehensive transcriptome using gffcompare software (version:0.9.8). After the final transcriptome was generated, StringTie and ballgown were used to estimate the expression levels of all transcripts and perform expression abundance for mRNAs by calculating FPKM value. Genes differential expression analysis was performed by DESeq2 software between two different groups (and by edgeR between two samples). The genes with the parameter of false discovery rate (FDR) below 0.05 and absolute fold change greater than or equal to 2 were considered differentially expressed genes. Differentially expressed genes were then subjected to enrichment analysis of gene ontology (GO) functions and Kyoto encyclopaedia of genes and genomes (KEGG) pathways. A ranked gene list obtained the differential expression analysis was input to ‘GSEA’ package for further analysis.

### 2.14. Statistical analysis

To calculate the WISH staining results, the embryos were analysed under a microscope after the staining and divided into two groups according to the staining results, a high-staining group and a low-staining group. For statistical analysis, groups with high staining were used in the *t*-test. Data of all triplicate experiments were reported as mean values ± s.d. Statistical analyses were performed using GraphPad Prism 8.0.2 (GraphPad Software, San Diego, CA, USA) using two-tailed student's *t*-tests. The Log-rank (Mantel-Cox) significance test was used to analyse survival curves. The statistical significance was displayed as ‘ns’ for no statistical significance, *p < .05, **p < .01 and ***p < .001.

## 3. Results

### 3.1. Overexpression of hoxa9 in blood cells inhibits myeloid development

**Figure 1.**
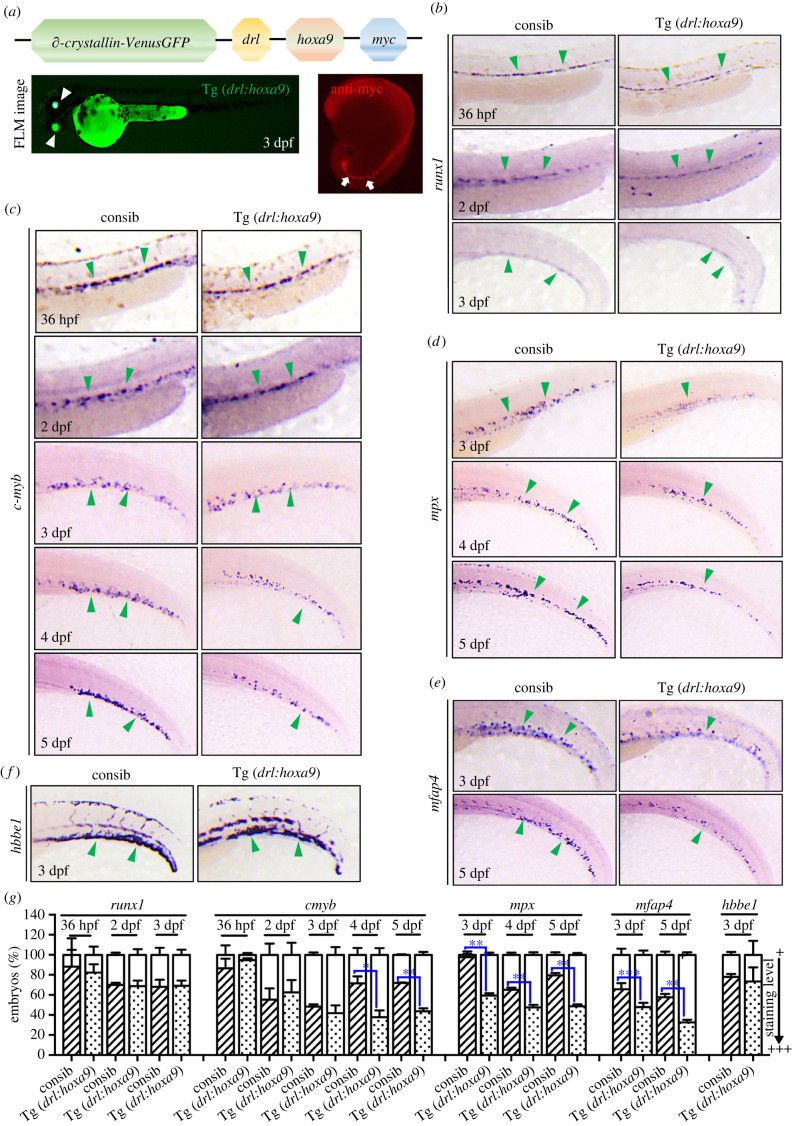
Transgenic zebrafish express *hoxa9* under the control of *drl* regulatory element. (*a*) The schematic representation of Tg(*drl*:*hoxa9*) expression with *∂*-crystallin::Venus GFP reporter gene. A GFP variant is expressed in the eyes under the control of the *∂*-crystallin lens promoter (white arrowheads). Whole-mount immunofluorescence staining with anti-myc antibody indicated the overexpression of *hoxa9* in blood cells (white arrows). (*b*) WISH assay for HSC gene, *runx1* in embryos at 36 hpf-3 dpf. (*c*) WISH for myeloid progenitor gene, *c-myb* in embryos at 36 hpf-5 dpf. (*d*) WISH for neutrophil gene, *mpx* in embryos at 3–5 dpf. (*e*) WISH for macrophage gene, *mfap4* in embryos 3–5 dpf. (*f*) WISH for erythroid gene, *hbbe1* in embryos 3 dpf. (*g*) Quantification of WISH results in (*b*–*f*) at 36 hpf-5 dpf (*n* = 50–200 embryos per group). Data are represented as mean ± s.d. *p < .05, **p < .01, ***p < .001. *drl, draculin*; FLM, fluorescence microscopy; WISH, whole-mount *in situ* hybridization; hpf, hours post-fertilization; dpf, days post-fertilization.

We first generated a transgenic line to overexpress *hoxa9* under the control of the zebrafish *drl* regulatory element. The *drl* elements are active in all lineages derived from the lateral plate mesoderm and thus covering all haematopoietic cells [25]. We cloned myc-tagged *hoxa9* into the plasmid which also contained an integration marker, gamma crystallin driving Venus GFP expression in lens [27]. The plasmid *∂-crystallin-VenusGFP-drl-hoxa9-myc* was injected into WT embryos at one-cell stage to generate a stable Tg(*drl*:*hoxa9*). At 3 dpf, GFP expression was observed in the eyes of transgenic embryos (figure 1*a*). Whole-mount immunofluorescence with anti-myc antibody revealed that *hoxa9* are indeed overexpressed in the blood cells in the transgene (figure 1*a*). We analysed the haematopoietic phenotypes in Tg(*drl*:*hoxa9*) embryos using WISH. Embryos overexpressing *hoxa9* did not affect the development of HSCs as indicated by the similar expression of *runx1* between WT and transgenic embryos from 36 h post-fertilization (hpf) to 3 dpf (figure 1*b*). Overexpressing *hoxa9* did not affect myeloid progenitor cell (*cmyb*) development at the early stages (36 hpf-3 dpf) but inhibited its formation at 4–5 dpf (figure 1*c*). In addition, Tg(*drl*:*hoxa9*) reduced the expression of the neutrophil marker *mpx* and the macrophage marker *mfap4* at 3–5 dpf, indicating that these embryos undergo myeloid development arrest (figure 1*d,e*). By contrast, Tg(*drl*:*hoxa9*) did not affect the expression of erythrocyte marker *hbbe1* at 3 dpf (figure 1*f*). Taken together, the overexpression of *hoxa9* in blood cells mainly inhibits myeloid development.

### 3.2. Induction of ubiquitous expression of MEIS1 does not affect embryonic haematopoiesis

**Figure 2.**
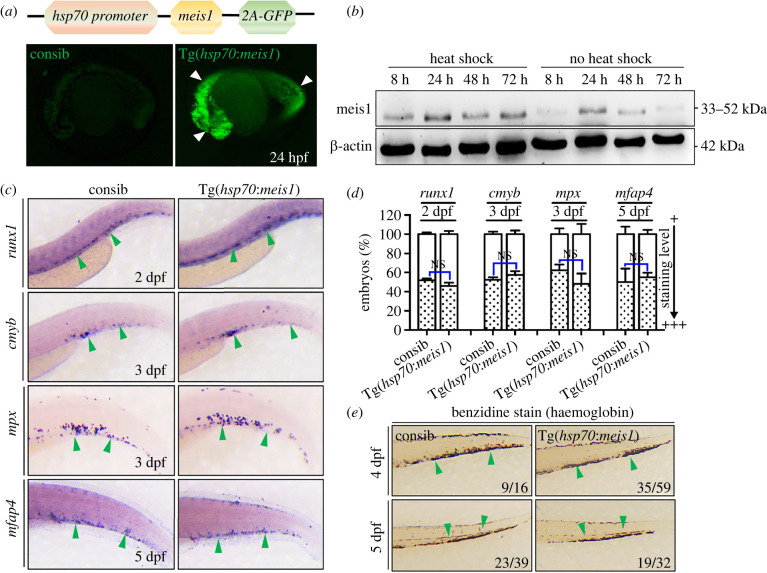
Transgenic zebrafish with inducible expression of *meis1* under the control of *hsp70* promoter. (*a*) The schematic representation of Tg(*hsp70*:*meis1*) expression vector. Ubiquitous GFP expression can be observed throughout whole embryos followed by HS for 1 h at 24 hpf (white arrowheads). (*b*) Western blot using a monoclonal anti-Meis1 antibody recognizes Meis1 protein at different times after HS induction. The antibody also recognizes endogenous Meis1 protein in the control group without HS. The mouse anti-β-actin antibody serves as controls. (*c*) Tg(*hsp70*:*meis1*) embryos were heat shocked at 24 hpf and assayed by WISH for *runx1* (2 dpf), *c-myb* (3 dpf), *mpx* (3 dpf) and *mfap4* (5 dpf) at different stages. (*d*) The quantification of WISH results in C (*n* = 50–100 embryos per group). Data are represented as mean ± s.d. NS means no statistical significance. (*e*) Benzidine staining of haemoglobin in mature erythrocytes in embryos at 4–5 dpf. HS, heat-shock. dpf, days post-fertilization.

Next, we generated a transgenic zebrafish to overexpress *meis1*. Zebrafish have 2 paralogues of *meis1*, *meis1a* and *meis1b*. *meis1b* shows 94% identity to its human counterpart at the amino acid level [31]. It is expressed throughout the embryos including haematopoietic cells and has been shown to regulate primitive and definitive haematopoiesis [32–34]. We cloned *meis1b* (hereafter *meis1*) under the control of the zebrafish *hsp70* promoter. The *pcs2-hsp70-meis1-2A-GFP* plasmid was injected into WT embryos at one-cell stage to generate the stable Tg(*hsp70*:*meis1*). At 24 hpf, the embryos were heat shocked at 38.5°C for 1 h, the transgenic embryos showed GFP expression throughout the whole body within a short time (figure 2*a*). Western blot experiments revealed that *meis1* protein was readily induced after heat shock and lasted at least for 72 h (figure 2*b*). We incubated WT and Tg(*hsp70*:*meis1*) embryos at 38.5°C for 1 h at 24 hpf, and fixed the embryos at 2–5 dpf for WISH. Embryos overexpressing *meis1* showed the similar levels of *runx1*, *cmyb*, *mpx* and *mfap4* at different stages compared to WT (figure 2*c*,*d*). In addition, benzidine-stained haemoglobin expression remained unaffected in Tg(*hsp70*:*meis1*) embryos after heat induction (figure 2*e*), indicating normal erythroid differentiation. Taken together, the overexpression of *meis1* throughout embryos alone did not influence embryonic haematopoiesis.

### 3.3. Co-overexpression of hoxa9 and meis1 inhibits myeloid, erythroid and lymphoid development and the formation of haematopoietic stem cells and myeloid progenitors

**Figure 3.**
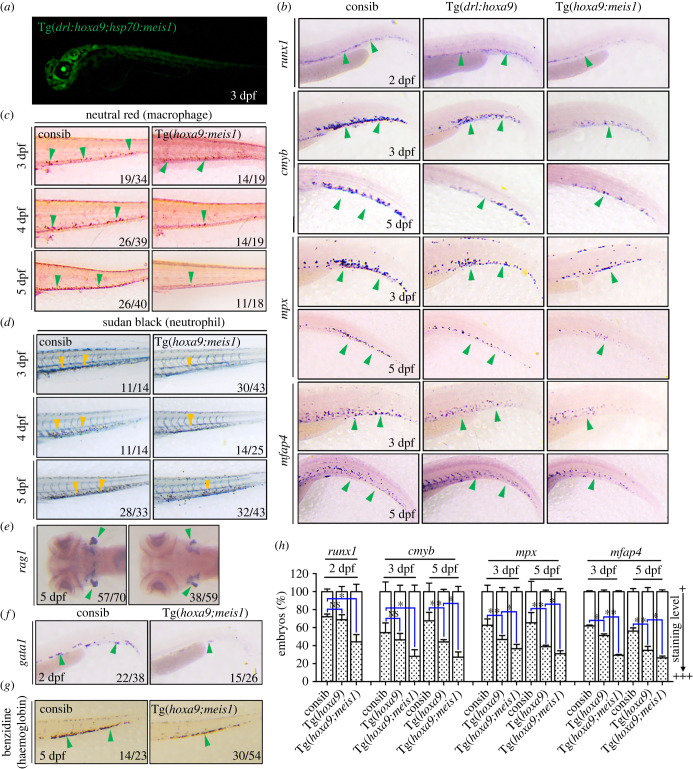
Tg(*drl*:*hoxa9*;*hsp70*:*meis1*) inhibits myeloid, erythroid and lymphoid differentiation and the formation of HSCs and myeloid progenitors. (*a*) Tg(*drl*:*hoxa9*;*hsp70*:*meis1*) was heat shocked at 24 hpf, and ubiquitous GFP expression was induced after HS. GFP was also expressed in the eyes under the control of the *∂*-crystallin promoter. (*b*) Tg(*drl*:*hoxa9*;*hsp70*:*meis1*) embryos and control siblings were heat shocked at 24 hpf and assayed by WISH for different genes in zebrafish haematopoiesis at different stages, *runx1* (2 dpf), *c-myb* (3 and 5 dpf), *mpx* (3 and 5 dpf) and *mfap4* (3 and 5 dpf). (*c*) Neutral red staining of macrophages in embryos at 3–5 dpf. (*d*) Sudan black staining of neutrophils in embryos at 3–5 dpf. (*e*) WISH assay for *rag1* at 5 dpf. (*f*) WISH assay for erythroid gene, *gata1* in embryos at 2 dpf. (*g*) Benzidine staining of haemoglobin in mature erythrocytes in embryos at 5 dpf. (*h*) The quantification of WISH results in (*b*) (*n* = 50–100 embryos per group). Data are represented as mean ± s.d. *p < .05, **p < .01. NS means no statistical significance. HS, heat-shock; dpf, days post-fertilization; WISH, whole-mount *in situ* hybridization.

We next crossed the two transgenic lines to establish a stable Tg(*drl*:*hoxa9*;*hsp70*:*meis1*) with inducible co-overexpression of *hoxa9* and *meis1* in blood cells (figure 3*a*). WT, Tg(*drl*:*hoxa9*) and Tg(*drl*:*hoxa9*;*hsp70*:*meis1*) embryos at 24 hpf were heat shocked at 38.5°C for 1 h and subjected to WISH at 2, 3 or 5 dpf. After induction, Tg(*drl*:*hoxa9*;*hsp70*:*meis1*) embryos significantly inhibited the development of *runx1*-labelled HSCs at 2 dpf, and the *cmyb*-labelled myeloid progenitor cells at 3 dpf compared to WT and Tg(*drl*:*hoxa9*) embryos. And Tg(*drl*:*hoxa9*;*hsp70*:*meis1*) after induction further decreased the formation of mature neutrophils (*mpx*) and macrophages (*mfap4*) that were already inhibited by Tg(*drl*:*hoxa9*) at 3 and 5 dpf (figure 3*b*). Neutral red staining of macrophages and Sudan black staining of neutrophils confirmed the strong inhibition of myeloid differentiation in Tg(*drl*:*hoxa9*;*hsp70*:*meis1*) embryos (figure 3*c*,*d*). Tg(*drl*:*hoxa9*;*hsp70*:*meis1*) also showed a decrease of lymphoid cells (*rag*1) at 5 dpf (figure 2*e*), a decrease of erythroid progenitors (*gata1*) at 2 dpf (figure 2*f*) and a decrease of mature erythrocytes (stained by Benzidine) at 5 dpf (figure 2*g*), indicating that Tg(*drl*:*hoxa9*;*hsp70*:*meis1*) also has defects in erythroid and lymphoid differentiation. To verify whether the phenotypes depend on the level of Meis1 protein induced by heat shock, we also heat-shocked Tg(*drl*:*hoxa9*;*hsp70*:*meis1*) embryos daily from 24 dpf and performed WISH assay at 5 dpf. The results showed that Tg(*drl*:*hoxa9*;*hsp70*:*meis1*) displayed similar haematopoietic defects under more frequent heat shock conditions (electronic supplementary material, figure S1). Together, these results showed that the co-overexpression of *hoxa9* and *meis1* in blood cells significantly inhibited the development of myeloid, erythroid and lymphoid cells, and the specification of haematopoietic stem and progenitor cells (HSPCs) compared to WT or Tg(*drl*:*hoxa9*) embryos. The lower production of mature blood cells coupled with poor HSPCs production in Tg(*drl*:*hoxa9*;*hsp70*:*meis1*) was reminiscent of the MDS phenotype [12].

### 3.4. Tg(drl:hoxa9;hsp70:meis1) adult fish displays acute myeloid leukaemia-like phenotype at 3 months old

To examine the possible development of leukaemia-like phenotype in adult fish, Tg(*drl*:*hoxa9*;*hsp70*:*meis1*) embryos and control sibling including WT, Tg(*drl:hoxa9*) and Tg(*hsp70*:*meis1*) were heat shocked once at 24 hpf for 1 h, and were then grown to adulthood. Most of the fish survived. Starting at 3 months old, the majority of Tg(*drl*:*hoxa9*;*hsp70*:*meis1*) zebrafish presented with abdominal masses compared to control siblings. The PB and KM cells were collected from the 3-month-old fish and subjected to May-Grünwald and Giemsa staining. The PB cells from the control were predominantly erythrocytes, with myelocytes only occasionally observed (electronic supplementary material, figure S2*a*,*b*). By contrast, Tg(*drl*:*hoxa9*;*hsp70*:*meis1*) fish contained noticeable myeloid precursor cells that have high nuclear to cytoplasmic ratio. These Tg(*drl*:*hoxa9*;*hsp70*:*meis1*) also demonstrated abundant myeloid precursors in the KM compared to WT, supporting the development of adult AML-like malignancies. These Tg(*drl*:*hoxa9*;*hsp70*:*meis1*) fish are now 1.5 years old, and many of them are still alive, although some are in a sick state.

**Figure 4.**
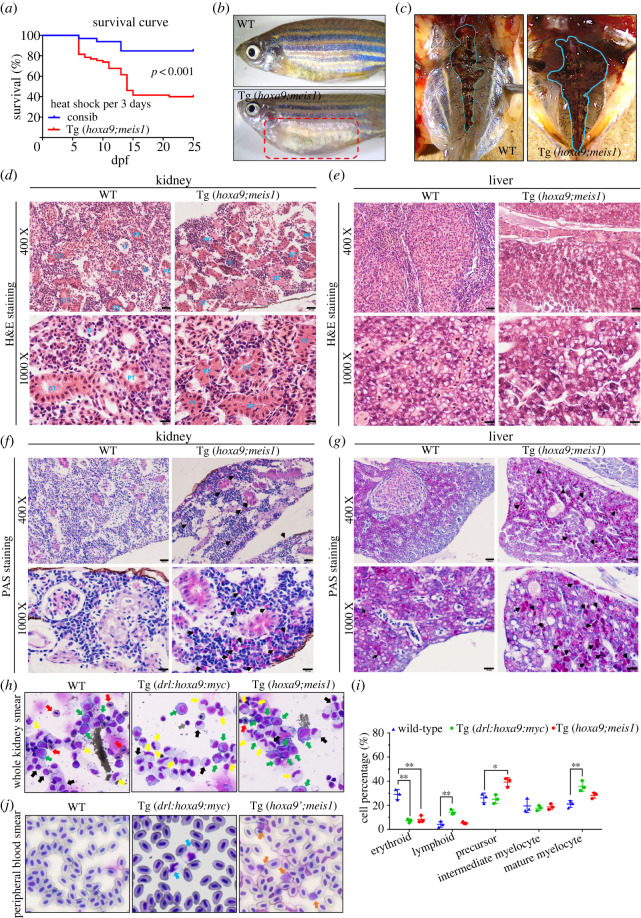
Tg(*drl*:*hoxa9*;*hsp70*:*meis1*) zebrafish develop disease similar to AML. (*a*) Survival curves of control and Tg(*drl*:*hoxa9*;*hsp70*:*meis1*) fish after heat-shock induction every 3 days starting at 24 hpf, *n* = 30–60, *p* < 0.001, Log-rank (Mantel-Cox) significance test. (*b*) Tg(*drl*:*hoxa9*;*hsp70*:*meis1*) embryos were heat shocked every 3 days starting at 24 hpf and then grown to adulthood. At 3 months old, many Tg(*drl*:*hoxa9*;*hsp70*:*meis1*) presented with abdominal masses and were susceptible to infection. (*c*) Gross anatomy of Tg(*drl*:*hoxa9*;*hsp70*:*meis1*) and WT kidneys (about 3 months old). (*d*,*e*) High-magnification imaging of H&E-stained tissue sections of WT and Tg(*drl*:*hoxa9/meis1*) kidneys and livers (400 x, scale bar = 20 µM and 1000 x, scale bar = 10 µM). The affected kidneys and livers showed the absence of normal tissue structures and presence of disorganized infiltrate. PT: proximal tubule, DT: distal tubule, G: glomerus. (*f*,*g*) High-magnification imaging of PAS-stained tissue sections of WT and Tg(*drl*:*hoxa9*;*hsp70*:*meis1*) kidneys and livers (400 x, scale bar = 20 µM and 1000 x, scale bar = 10 µM). Black arrows indicate PAS ^±^ -granulocytic myeloid cells. (*h*) May-Grünwald and Giemsa staining of the whole KM of 3-month-old WT, Tg(*drl*:*hoxa9*) and Tg(*drl*:*hoxa9*;*hsp70*:*meis1*). The expanded precursors were detected in Tg(*drl*:*hoxa9*;*hsp70*:*meis1*) (green arrows). Red arrows indicate erythroid cells. Grey arrows indicate lymphoid cells. Black arrows indicate mediate-differentiation cells. Yellow arrows indicate fully differentiation cells. (*i*) Statistical analysis of various haematopoietic cells from whole KM of WT, Tg(*drl*:*hoxa9*) and Tg(*drl*:*hoxa9*;*hsp70*:*meis1*) fish. (*j*) May-Grünwald and Giemsa staining of the PB smear of 3-month-old WT, Tg(*drl*:*hoxa9*) and Tg(*drl*:*hoxa9*;*hsp70*:*meis1*). Blue arrows indicate the infiltration of mature myeloid cells into PB in Tg(*drl*:*hoxa9*). Orange arrows indicate the infiltration of myelomonocytes and precursors into PB in Tg(*drl*:*hoxa9*;*hsp70*:*meis1*). Data are represented as mean ± s.d. *p < .05, **p < .01, ***p < .001. H&E, haematoxylin & eosin; PAS, periodic acid-Schiff.

To enhance the adult myeloid malignancies, Tg(*drl*:*hoxa9*;*hsp70*:*meis1*) embryos and their siblings were heat shocked once at 24 hpf, followed by continuous heat shock pulse treatment every 3 days as induced Meis1 protein lasts for at least 3 days. Under this condition, Tg(*drl*:*hoxa9*;*hsp70*:*meis1*) fish died rapidly starting from 6 dpf, significantly higher than control siblings (figure 4*a*), which is similar to the rapid death observed in mouse recipients of bone marrow cells overexpressing *Hoxa9* and *Meis1* [24,35]. About 20–40% of embryos survived to adulthood. At three-month old, almost all Tg(*drl*:*hoxa9*;*hsp70*:*meis1*) presented with abdominal masses and were susceptible to infection compared to control fish (figure 4*b*), and these Tg(*drl*:*hoxa9*;*hsp70*:*meis1*) fish died within 6 months after birth. We dissected the three-month-old fish and performed H&E staining on tissue sections. Tg(*drl*:*hoxa9*;*hsp70*:*meis1*) demonstrated a significantly enlarged kidney compared to the control (figure 4*c*). The normal zebrafish kidney is composed of nephron functional units in arborized arrangements, surrounded by haematopoietic tissue that is dispersed throughout the intervening spaces (figure 4*d*). By contrast, Tg(*drl*:*hoxa9*;*hsp70*:*meis1*) showed infiltration of cells throughout the intervening spaces (figure 4*d*). Moreover, Tg(*drl*:*hoxa9*;*hsp70*:*meis1*) also showed invaded cells in the liver (figure 4*e*). In addition, we confirmed that a subset of infiltrating cells in kidney or liver were myeloid cells using PAS staining (figure 4*f*,*g*). To monitor blood composition, we performed the cytological analysis on the whole KM cells from WT, Tg(*drl*:*hoxa9*) and Tg(*drl*:*hoxa9*;*hsp70*:*meis1*) adult fish. Compared to WT, Tg(*drl*:*hoxa9*) showed a strong increase of metamyelocyte population. By contrast, Tg(*drl:hoxa9*;*hsp70*:*meis1*) showed a strong expansion of myeloid precursors, along with an inhibition of myeloid differentiation (figure 4*h–j*). The expanded immature precursors in Tg(*drl*:*hoxa9*;*hsp70*:*meis1*) exhibited large nuclei, high karyoplasmic ratio and intensive cytoplasmic staining, resembling the AML blasts (figure 4*h*–*j*). In addition, flow cytometry analysis of KM also revealed that the percentage of immature precursors was increased and the myelomonocyte populations were decreased in Tg(*drl*:*hoxa9*;*hsp70*:*meis1*) (electronic supplementary material, figure S3). Moreover, both Tg(*drl:hoxa9*) and Tg(*drl*:*hoxa9*;*hsp70*:*meis1*) exhibited a decrease of the erythrocyte population, indicating anaemia in these fish. We also performed cytological analysis of PB cells, and the results revealed that compared to WT, the PB of Tg(*drl*:*hoxa9*) contained noticeable mature myelocytes (figure 4*j*), which was less common at 3 months old but became common at 12-month old. By contrast, the PB of Tg(*drl*:*hoxa9*;*hsp70*:*meis1*) showed a significantly increased presence of poorly differentiated precursors at the age of 3 months (figure 4*j*). The accumulation of immature myeloid precursor cells and interrupted myeloid and erythroid differentiation in the KW and the infiltration of myeloid precursors into the circulation in Tg(*drl*:*hoxa9*;*hsp70*:*meis1*) fish are reminiscent to the symptoms of human AML, supporting that Tg(*drl*:*hoxa9*;*hsp70*:*meis1*) after induction rapidly develops an AML-like malignant myeloid disease in adulthood.

### 3.5. Genome-wide transcriptional changes in kidney marrow cells in Tg(drl:hoxa9;hsp70:meis1)

**Figure 5.**
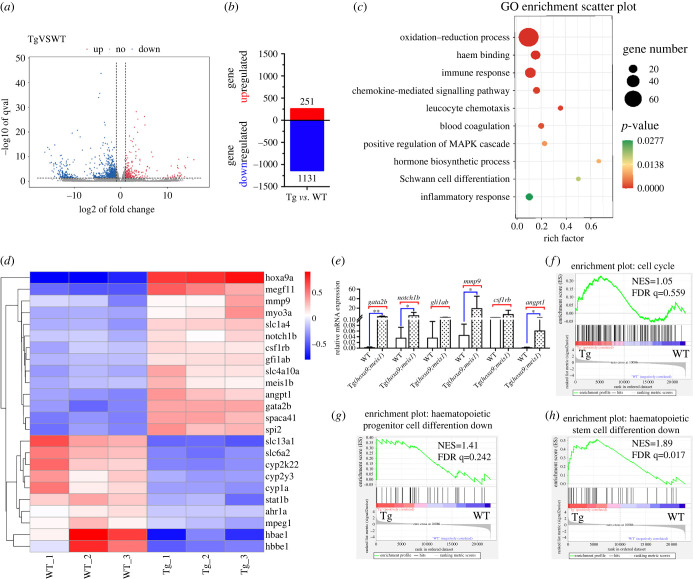
Genome-wide transcriptional analysis of the KM cell of WT and Tg(*drl*:*hoxa9*;*hsp70*:*meis1*) adult fish. (*a*) The volcano plot for differential gene expression between WT and Tg(*drl*:*hoxa9*;*hsp70*:*meis1*) from three biological replicates. (*b*) Numbers of up- and downregulated genes in Tg(*drl*:*hoxa9*;*hsp70*:*meis1*) relative to WT (greater than twofold change, adj *p* ≤ 0.05). (*c*) GO enrichment analysis of differential expressed genes. (*d*) Heatmap display of the representative differentially expressed genes between WT and Tg(*drl*:*hoxa9*;*hsp70*:*meis1*). (*e*) qPCR assay on the mRNA levels of a number of genes in KW cells in Tg(*drl*:*hoxa9*;*hsp70*:*meis1*) compared to WT. Data are represented as mean ± s.d. *p < .05, **p < .01. (*f*–*h*) GSEA of the expressing profile of KM cells in WT and Tg(*drl*:*hoxa9*;*hsp70*:*meis1*) using cell cycle-associated upregulated signature, haematopoietic progenitor cell differentiation-associated downregulated signature and HSC differentiation-associated downregulated signature. KW, kidney marrow.

To further characterize the haematopoietic defects and identify the transcription activity induced by co-overexpression of *hoxa9* and *meis1*, we performed RNA-sequencing analysis of the WKM cells from three-month-old WT and Tg(*drl*:*hoxa9*;*hsp70*:*meis1*) fish. We found 251 genes upregulated and 1131 genes downregulated in WKM cells in Tg(*drl*:*hoxa9*;*hsp70*:*meis1*) (FDR < 0.05 and greater than twofold change, figure 5*a*,*b*). The GO enrichment analysis showed that signalling pathways including oxidation–reduction process, immune response, chemokine-mediated signalling pathway, leucocyte chemotaxis blood coagulation, haem binding and positive regulation of MAPK cascade were enriched in Tg(*drl*:*hoxa9*;*hsp70*:*meis1*) (figure 5*c*). In the differentially expressed genes, genes expected to be enriched like *hoxa9a* (fold change, fc = 7.74) and *meis1b* (fc = 2.46) were indeed highly unregulated. The top upregulated genes included *gata2b* (fc = 7.35), *notch1b* (fc = 2.07), *gfi1ab* (fc = 2.25) and *mmp9* (fc = 2.36), which are required for maintenance and/or expansion of HSCs and multi-potent progenitors, or strongly implicated in leukaemia transformation [36–44]. Other significantly upregulated genes included *angpt1*, *csf1rb*, *myo3a*, *megf11*, *slc4a10a* and *spaca4l* that have been shown to be overexpressed in AMLs from previous studies [45,46] or according to the analysis from Bloodspot database (www.bloodspot.eu). *spi2* expression was also strongly upregulated (fc = 6.44), which is required for cell differentiation and transcription [47]. The strikingly downregulated genes in Tg(*drl*:*hoxa9*;*hsp70*:*meis1*) included *mpeg1* (fc = 0.44) and *hbae1* (fc = 0.04), supporting the inhibition of haematopoietic differentiation. *ahr1a* and *ahr1b* were also significantly downregulated in Tg(*drl*:*hoxa9*;*hsp70*:*meis1*), consistent with the previous finding showing that diminished AHR signalling drives human AML stem cell maintenance [48,49]. Other downregulated genes were *tmem88a*, *trim2a*, *ephB4*, *jag2b*, *notch3* and *unc5b*, which are known to regulate vascular development [50]. We validated the expression of the representative genes by qPCR assay (figure 5*e*). In line with the finding, the gene set enrichment analysis (GSEA) revealed positive enrichment in Tg(*drl*:*hoxa9*;*hsp70*:*meis1*) for the gene signature with cell cycle, indicating the increased cell proliferation (figure 5*f*), and with the downregulated genes in haematopoietic progenitor cell differentiation as well as HSC differentiation (figure 5*g*,*h*), indicating a differentiation blockade. Thus, gene expression signature of WKM cells in Tg(*drl*:*hoxa9*;*hsp70*:*meis1*) verified the AML-like myeloid malignant phenotype. Interestingly, KEGG enrichment analysis identified many amino acid metabolic pathways significantly overexpressed in Tg(*drl*:*hoxa9*;*hsp70*:*meis1*), such as tryptophan metabolism, glycine and serine, glutamate, and arginine metabolism (electronic supplementary material, figure S4), indicating that the dysregulated amino acid metabolism is probably involved in the myeloid malignancies in Tg(*drl*:*hoxa9*;*hsp70*:*meis1*), which is consistent with recent studies showing the elevated amino acid metabolism in leukaemia stem cells and primary blasts [51,52].

### 3.6. Tg(drl:hoxa9;hsp70:meis1) acute myeloid leukaemia model responds to leflunomide treatment

**Figure 6.**
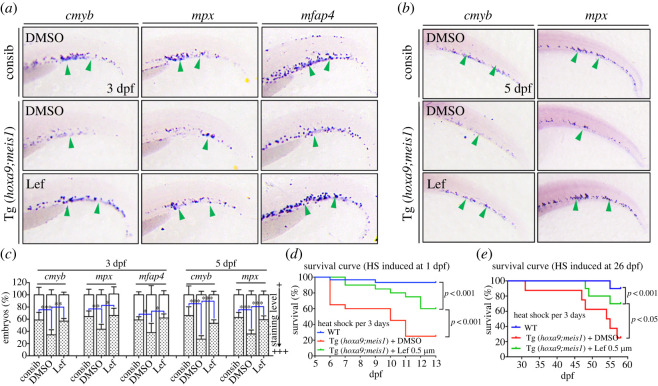
DHODH inhibitor leflunomide rescues defective haematopoiesis and improves the survival in Tg(*drl*:*hoxa9*;*hsp70*:*meis1*) fish. (*a*,*b*) Tg(*drl*:*hoxa9*;*hsp70*:*meis1*) embryos and control siblings were heat-shock induced at 24 hpf. Tg(*drl*:*hoxa9*;*hsp70*:*meis1*) embryos were treated with DMSO or Lef after heat shock. Embryos were assayed by WISH for the expression of *cmyb*, *mpx* and *mfap4* at 3 dpf or 5 dpf. (*c*) Quantification of WISH results in (*a*,*b*) (*n* = 50–100 embryos per group). Data are represented as mean ± s.d. *p < .05, **p < .01, ***p < .001. (*d*) WT and Tg(*drl*:*hoxa9*;*hsp70*:*meis1*) were repeatedly heat-shock induced starting from 24 hpf, Tg(*drl*:*hoxa9*;*hsp70*:*meis1*) fish were treated with DMSO or Lef after the initial heat shock. Survival curves of WT and Tg(*drl*:*hoxa9*;*hsp70*:*meis1*) fish (*n* = 20–30 per group, Log-rank (Mantel-Cox) significance test). (*e*) WT and Tg(*drl*:*hoxa9*;*hsp70*:*meis1*) were repeatedly heat shock induced starting from 26 dpf, Tg(*drl*:*hoxa9*;*hsp70*:*meis1*) fish were treated with DMSO or Lef after the initial heat shock. Survival curves of WT and Tg(*drl*:*hoxa9*;*hsp70*:*meis1*) fish (*n* = 10 per group, Log-rank (Mantel-Cox) significance test). Lef, Leflunomide; hpf, hours post-fertilization; dpf, days post-fertilization.

We next examined the effects of anti-leukaemic agent in Tg(*drl*:*hoxa9*;*hsp70*:*meis1*) zebrafish. Recent studies have identified DHODH as a therapeutic target for AML, and DHODH inhibitors cause myeloid differentiation and show potent anti-leukaemia effects in several AML mouse models including the Hoxa9/Meis1 model [2]. Tg(*drl*:*hoxa9*;*hsp70*:*meis1*) embryos were heat shocked at 24 hpf and then treated with Leflunomide (Lef), a DHODH inhibitor active in zebrafish [53]. The haematopoietic phenotypes were examined at 3 dpf or 5 dpf by WISH. Lef treatment significantly rescued the number of *c-myb*^+^ myeloid progenitors, *mpx*^+^ neutrophils and *mfap4*^+^ macrophages at 3 dpf and 5 dpf in Tg(*drl*:*hoxa9*;*hsp70*:*meis1*) embryos compared to the embryos without treatment (figure 6*a*–*c*), supporting the ability of Lef to promote myeloid differentiation in zebrafish. To further verify if Lef induces myeloid differentiation, we analysed the PB from 7-dpf Tg(*drl:hoxa9;hsp70:meis1*) fish after induction with and without Lef treatment. The cytology analysis revealed an increase of differentiated granulocytes in a subset of Lef-treated transgenic embryos compared to untreated controls (electronic supplementary material, figure S5), supporting that Lef could induce a mild differentiation in some Tg(*drl:hoxa9;hsp70:meis1*) fish.

To further evaluate the anti-leukaemia activities of Lef, Tg(*drl*:*hoxa9*;*hsp70*:*meis1*) embryos were heat induced at 24 hpf and followed by heat treatment every 3 days. As expected, Tg(*drl*:*hoxa9*;*hsp70*:*meis1*) died quickly starting from 6 dpf. Lef treatment significantly prolonged and increased the survival of these embryos (figure 6*d*). To examine whether Lef could rescue Tg(*drl*:*hoxa9*;*hsp70*:*meis1*) embryos induced at a later stage, the fish were heat shocked at 26 dpf and following by treatment every 3 days. Most transgenic fish died starting from 46 dpf, and Lef treatment also improved the survival rate of these fish (figure 6*e*). Collectively, the results supported that Lef played a conserved function to promote myeloid differentiation and inhibit leukaemogenesis in Tg(*drl*:*hoxa9*;*hsp70*:*meis1*). The conserved pharmacological mechanism in transgenic zebrafish and mouse model supports that Tg(*drl*:*hoxa9*;*hsp70*:*meis1*) is a promising model for AML drug discovery.

## 4. Discussion

AML occurs when multiple genetic aberrations alter blood cell development, leading to hyperproliferation and arrest of cell differentiation [15]. Animal model is crucial to study the underlying mechanism and discover novel therapies. Although murine AML models have been widely used, they have several major limitations such as high cost, time-consuming and difficult to perform high-throughput screening. In this study, transgene-driving expression of *hoxa9* and *meis1* in zebrafish strongly perturbed the development of blood cells in early embryos and efficiently induced AML-like phenotypes at 3 months old. Our results support that zebrafish Tg(*drl*:*hoxa9*;*hsp70*:*meis1*) is a novel rapid-onset high-penetrance model for human AML.

Previous studies have established different myeloid leukaemia models using zebrafish [10–12]. Most of these models display embryonic haematopoietic defects and recapitulate partial AML phenotypes. Much has been discovered regarding mechanisms underlying these AML-like phenotypes. But these zebrafish myeloid malignancy models frequently fail to develop the full adult disease state either due to embryonic lethality or the long latency and low penetrance [9]. *Hoxa9*/*Meis1* combination is among the most potent AML-inducing oncogenes that induce the fastest occurrence of leukaemia in mice [22]. In our studies, we developed a germline transgenic zebrafish harbouring *hoxa9*/*meis1*. The heat shock induction strategy confers a degree of experimental control that robustly induces co-overexpression of *hoxa9*/*meis1* in blood cells upon heat treatment. During embryogenesis, with only one time of heat shock, Tg(*drl*:*hoxa9*;*hsp70*:*meis1*) embryos demonstrated reduced myeloid and erythroid differentiation and poor development of HSPCs, reminiscent of the MDS phenotype. The one-time heat-treated Tg(*drl*:*hoxa9*;*hsp70*:*meis1*) fish survived to adults and developed to adult AML-like malignancy at 3 months old. Tg(*drl*:*hoxa9*;*hsp70*:*meis1*) larvae received repeated heat shock treatments from the early developmental stage died rapidly at 6–15 dpf. A subgroup of these fish survived and developed adult AML readily at 3 months old with almost 100% penetrance. These fish showed expansion of immature myeloid precursors, interrupted differentiation and anaemia in the KM and infiltration of immature precursors into the PB, which resembles the human AML. In order to determine the aggressiveness of the leukaemia induced by Tg(*drl:hoxa9;hsp70:meis1*), future studies will need to examine if the AML-like phenotype developed here could be transplanted into WT adult hosts by whole KM cell transplantation. Our results also indicated that the acceleration of AML by *meis1* depends on the expression level of Meis1 protein, which is consistent with the previous studies showing that MEIS1 expression level is inversely correlated with the prognosis of human AML [21]. In addition, we also heat-treated adult Tg(*drl*:*hoxa9*;*hsp70*:*meis1*), and the transgenic fish died rapidly within two weeks, supporting that induction at the adult stage also potentially induces AML. More importantly, DHODH inhibitor, which causes myeloid differentiation and inhibits leukaemogenesis in murine AML models, also restored haematopoiesis in Tg(*drl*:*hoxa9*;*hsp70*:*meis1*) embryos and improved their late survival. Thus, zebrafish Tg(*drl*:*hoxa9*;*hsp70*:*meis1*) displays similar myeloid malignant phenotypes and pharmacological mechanism to murine *Hoxa9*/*Meis1* AML model. It is an attractive AML model and is valid for studying AML pathogenesis and evaluating anti-leukaemic drugs.

HOXA9 is overexpressed in about 80% of AML. Hoxa9 overexpression alone in mouse bone marrow leads to a myeloproliferative disorder. A subgroup of these animal progresses to AML around 7 months. In zebrafish, overexpressing *hoxa9* in blood cells by Tg(*drl*:*hoxa9*) induces myeloid differentiation arrest in embryos and leads to the expansion of myelocyte populations in the KM and PB in adulthood, resembling the phenotype of MPN. By contrast, *meis1* overexpression in whole zebrafish did not show noticeable effects on haematopoiesis, consistent with the findings from mouse studies, in which *Meis1*-transduced cells do not develop haematopoietic malignancies after a long period of observation [24]. Compared to Tg(*drl*:*hoxa9*), Tg(*drl*:*hoxa9*;*hsp70*:*meis1*) further decreased myeloid differentiation and caused anaemia in embryos and expanded immature myeloid precursors and interrupted differentiation in adults. Our results supported the conserved and potent functions of *meis1* in dramatically accelerating *hoxa9*-induced leukaemias in zebrafish, also suggested that *meis1* may primarily function to block the terminal differentiation of myeloid cells transduced by *hoxa9*. Interestingly, in *NUP98*-*HOXA9* harbouring patients, *MEIS1* was found to be upregulated. It is also reported that Tg(*NUP98*-*HOXA9*) zebrafish expands haematopoietic progenitor cells depending on downstream activation of *meis1* [15]. Thus, Meis1 might play a more general role in the leukaemic transformation of myeloid cells induced by different factors such as *NUP98*-*HOXA9*.

Both *Hoxa9* and *Meis1* are preferentially expressed in primitive haematopoietic cells and are downregulated during differentiation [16,21]. The overexpression of *Hoxa9* and *Meis1* flip the cell into a self-perpetuating proliferating state [16]. The Hox-homeodomain determines binding specificity Hoxa9 and the remaining portions interact with cofactors, such as the Meis1, which further increases DNA-binding affinity and specificity [23]. Several studies have characterized the target genes regulated by Hoxa9 or Meis1, and revealed a limited number of targets. But our understanding of their mechanisms remains incomplete. The few leukaemic clones observed in murine Hoxa9/Meis1 models suggest that the transformation occurred in a rare cell type which is less abundant than the majority of the clonogenic progenitors [24]. Future studies by directly comparing the target genes in Tg(*drl*:*hoxa9*) and Tg(*drl*:*hoxa9*;*hsp70*:*meis1*) fish plus single-cell-transcriptome analysis will help reveal the detailed mechanism of the subclones transformed by Hoxa9/Meis1. Interestingly, Tg(*drl*:*hoxa9*;*hsp70*:*meis1*) embryos demonstrate the MDS-like phenotype. In human, up to 30% of MDS progresses to AML [54]. It is also worth studying the mechanisms by which embryonic MDS progresses to adult AML in Tg(*drl*:*hoxa9*;*hsp70*:*meis1*). Moreover, our comparative transcriptomic analysis of KM cells revealed an enrichment in amino acid metabolism in Tg(*drl*:*hoxa9*;*hsp70*:*meis1*). Recent studies have found that metabolism of specific amino acids including cysteine, glutamine and branched chain amino acids are important in multiple myeloid malignancies, and pharmacological targeting of these processes has shown therapeutic effects [52]. Considering that both Hoxa9 and Meis1 are currently considered undruggable, it is worth further deciphering the relationships between amino acid metabolism and leukaemogenesis in the KM to help develop amino acid disruption strategies for AML treatment.

Altogether, our data described that Tg(*drl*:*hoxa9*;*hsp70*:*meis1*) develops an AML-like myeloid malignant disease. This model confirms the potent transforming properties of Hoxa9/Meis1 observed in murine models. It provides a novel tool for elucidating the molecular pathogenesis of the transformation by *Hoxa9/Meis1* and is suitable for phenotype-based small molecule screen to identify potential agents for the treatment of AML.

## Data Availability

Raw data from the RNA-seq analysis have been deposited in NCBI Gene Expression Omnibus (GEO) with the accession number GSE204744. The data are provided in the electronic supplementary material [55].
